# IS THERE A WORRY ADVANTAGE? THE ROLE OF FEAR OF LONELINESS IN OLD AGE IN LONELINESS PREVENTIVE ACTIVITIES

**DOI:** 10.1093/geroni/igad104.0896

**Published:** 2023-12-21

**Authors:** Nora Degen, Yaeji Kim-Knauss, Frieder Lang

**Affiliations:** Friedrich-Alexander-Universität Erlangen-Nürnberg, Nürnberg, Bayern, Germany; Friedrich-Alexander-Universität Erlangen-Nürnberg, Nuremberg, Bayern, Germany; Friedrich-Alexander-Universität Erlangen-Nürnberg, Nuremberg, Bayern, Germany

## Abstract

While worries are generally known to be aversive, behavioral theories have also addressed potential advantages of worrying throughout life. Drawing on this notion, the present study focused on the fear of loneliness in old age and its beneficial effects on individuals’ engagement in loneliness preventive activities. Data were collected from 702 German adults (aged 22-96) who had participated in at least two out of five measurement occasions with a time interval of two years. Fear of loneliness in old age was measured using a single item (i.e., how much do you fear being lonely in old age?) and loneliness preventive activities were measured as a mean score of five social activities specifying the very intention to prevent loneliness in old age (e.g., I actively pursued contacts with friends and acquaintances in order not to be lonely in old age). Based on longitudinal multilevel regression models, we found that at the between-person level old age, female gender, and better health were independently associated with higher average loneliness preventive activities. At the within-person level, participants reported more preventive activities on occasions when they were more feared for the loneliness in old age. Such benefits of fear were particularly reported on occasions when individuals reported less state loneliness at that time. Findings indicate that aging-related worry serves as a motivator for loneliness preventive activities, particularly in situations in which individuals felt socially well connected.

